# Efficiency of Sucrose to Starch Metabolism Is Related to the Initiation of Inferior Grain Filling in Large Panicle Rice

**DOI:** 10.3389/fpls.2021.732867

**Published:** 2021-09-13

**Authors:** Zhengrong Jiang, Qiuli Chen, Lin Chen, Hongyi Yang, Meichen Zhu, Yanfeng Ding, Weiwei Li, Zhenghui Liu, Yu Jiang, Ganghua Li

**Affiliations:** ^1^College of Agronomy, Nanjing Agricultural University, Nanjing, China; ^2^Key Laboratory of Crop Physiology Ecology and Production Management, Ministry of Agriculture, Nanjing, China; ^3^Jiangsu Collaborative Innovation Center for Modern Crop Production, Nanjing, China; ^4^National Engineering and Technology Center for Information Agriculture, Nanjing, China

**Keywords:** rice, grain filling, inferior spikelets, initiation, sucrose, metabolism

## Abstract

The poor grain-filling initiation often causes the poor development of inferior spikelets (IS) which limits the yield potential of large panicle rice (*Oryza sativa* L.). However, it remains unclear why IS often has poor grain-filling initiation. In addressing this problem, this study conducted a field experiment involving two large panicle rice varieties, namely CJ03 and W1844, in way of removing the superior spikelets (SS) during flowering to force enough photosynthate transport to the IS. The results of this study showed that the grain-filling initiation of SS was much earlier than the IS in CJ03 and W1844, whereas the grain-filling initiation of IS in W1844 was evidently more promoted compared with the IS of CJ03 by removing spikelets. The poor sucrose-unloading ability, i.e., carbohydrates contents, the expression patterns of *OsSUTs*, and activity of CWI, were highly improved in IS of CJ03 and W1844 by removing spikelets. However, there was a significantly higher rise in the efficiency of sucrose to starch metabolism, i.e., the expression patterns of *OsSUS4* and *OsAGPL1* and activities of SuSase and AGPase, for IS of W1844 than that of CJ03. Removing spikelets also led to the changes in sugar signaling of T6P and SnRK1 level. These changes might be related to the regulation of sucrose to starch metabolism. The findings of this study suggested that poor sucrose-unloading ability delays the grain-filling initiation of IS. Nonetheless, the efficiency of sucrose to starch metabolism is also strongly linked with the grain-filling initiation of IS.

## Introduction

Rice (*Oryza sativa* L.) is one of the most important food crops in the world. Due to this, it is significant to increase rice yield and quality to meet the growing demand (Khush, 2005; Fageria, 2007). Rice yield is determined by panicle numbers, spikelet numbers per panicle, and grain-filling quality (Kato et al., 2015). The rice panicle of cultivating large-panicle varieties is composed of a large number of spikelets, and each spikelet is meaningful to produce high-quality grain at maturity (Peng et al., 1999). Some studies have found that the large panicle rice cultivars frequently fail to reach their high yield potential due to their poor grain-filling in inferior spikelets (IS) (Jun et al., 2008; Yang and Zhang, 2010). However, recent literature has shown that the slow grain-filling problem of IS was limited by the long lag phase and poor initiation of grain filling in IS of rice (Zhang et al., 2015; Das et al., 2016; Chen et al., 2019a). Most of the superior spikelets (SS) in the primary apical branches initiate and grow faster to achieve higher final dry weight at maturity, but the IS of the basal secondary branches of the panicle has a long developmental stagnancy stage after flowering (Zhou et al., 1992; Ishimaru et al., 2003). The long lag phase of IS postpones the grain development until the nutrients portioned in it are likely to be enough (Chen et al., 2019a). However, this is not good for the grain-filling initiation of IS. Previous studies always focused on the poor grain-filling of IS, but few have provided insights into its poor grain-filling initiation in large panicle rice.

Grain-filling initiation is determined by a complex mechanism. The phenomenon of poor grain-filling initiation is similar to those species with different arrangements of seeds, such as maize (Abrecht and Carberry, 1993; Yu et al., 2017) and wheat (Liang et al., 2017; Wang et al., 2017). The grain-filling initiation process is an interacting process of sink-source-flow (Zhao et al., 2006). Some researchers have found that the low availability of soluble carbohydrates is the main factor that causes the delayed growth of maize kernels (Shen et al., 2018). Changing the supply of soluble carbohydrates showed different influences on the grain-filling of IS in various types of rice (You et al., 2016; Chen et al., 2019a; Deng et al., 2021). However, the process of assimilate supply in IS is still unknown during the grain-filling initiation period. The sucrose is unloaded from the phloem of grains, which is used to supply soluble carbohydrates for the grain-filling process (Wu et al., 2016). The process of sucrose unloading plays a pivotal role in carbohydrates partitioning and the accumulation of sugars in the sink organs (Chen et al., 2017; Deng et al., 2021). The sucrose-proton symporter (SUT) and cell wall invertase (CWI) are crucial for sucrose unloading in developing spikelets (Lim et al., 2006; Bihmidine et al., 2013; Braun et al., 2014). The SUT coding genes, namely *OsSUT1* and *OsSUT2*, have been identified in grains of rice (Naohiro et al., 2003). Additionally, the CWI is the key regulator for the sucrose hydrolysis and the release of hexoses in rice (Braun et al., 2014; Chen et al., 2019a). Therefore, the question of whether increasing the sucrose-unloading will improve the inferior grain-filling initiation and the mechanism for that is needed to be answered.

Once the soluble carbohydrates reach the spikelet following phloem unloading, the soluble carbohydrates will go through various complex processes of metabolic, biosynthetic, or signaling processes (Braun et al., 2014). Grain-filling initiation is a process of metabolism from sucrose to starch through a series of enzymatically catalyzed reactions. (Bahaji et al., 2014; Dong and Beckles, 2019). It is generally accepted that sucrose synthase (SuSase) and adenosine diphosphate (ADP)-glucose pyrophosphorylase (AGPase) are thought to play an essential role in the metabolism of sucrose to starch in rice (Zhang et al., 2011; Ragel et al., 2013; Fan et al., 2019). Furthermore, the expression of *OsSuS4* and *OsAGPL1* is involved in regulating the activity of SuSase and AGPase (Cheng et al., 2015; Meng et al., 2020). Some researchers have found that the starch synthesis, key enzymes, and key gene expression are all involved in the metabolism of sucrose to starch (Wang et al., 2014). Interestingly, sucrose can act as molecular signals in response to starch synthesis in grains; sucrose signals increased grain yield by improving sucrose metabolism in grains (Chen et al., 2019b; Li et al., 2020). Sugar signaling can respond to sucrose supply to support grain growth (Martinez-Barajas et al., 2011). Moreover, the acclimation of sink-limited growth conditions can be altered by the sugar signal trehalose 6-phosphate (T6P) and the protein kinase (SnRK1) (Nunes et al., 2013). Some recent studies suggested that sugar signals have been shown to be necessary and sufficient for regulating the initial outgrowth and sugar metabolism of axillary bud (Mason et al., 2014; Wang et al., 2021). Generally, the regulation sugar signaling of T6P exists in sink tissues for activating starch synthesis and accumulation (Griffiths et al., 2016; Ponnu et al., 2020). Additionally, the sugar signaling of T6P has been found to be associated with the activation of AGPase for starch synthesis in leaves (Lunn et al., 2006; Ceusters et al., 2019). A recent study on pea has established that the sugar signaling of SnRK1 is involved in the response of early cotyledon establishment and patterning (Radchuk et al., 2010). In addition, the SnRK1 (*OsSnRK1a*) negatively regulates the growth and development of rice (Filipe et al., 2018). Nevertheless, there is no combined analysis of sugar signaling in T6P and SnRK1 that has yet been performed in grain-filling initiation of IS in rice. Thus, the relationship between sugar signaling, metabolic competence of sucrose to starch, and poor grain-filling initiation in IS remains unclear.

The objective of this study was to investigate whether the sucrose-unloading level, the metabolism process of sucrose to starch, and the regulation of sugar signaling were the limiting mechanism of IS grain-filling initiation in large panicle rice. Based on the strategy of removing SS in panicles, we examined the seed setting rate, grain weight, grain filling rate, starch content, sucrose-unloading ability, metabolism of sucrose to starch. This study further examined the T6P/SnRK1 pathway in the IS during the grain-filling initiation period. This study provided the first insights into the complex role of the limiting mechanism in IS grain-filling initiation in large-panicle rice.

## Materials and Methods

### Plant Materials and Management

**Figure 1:**
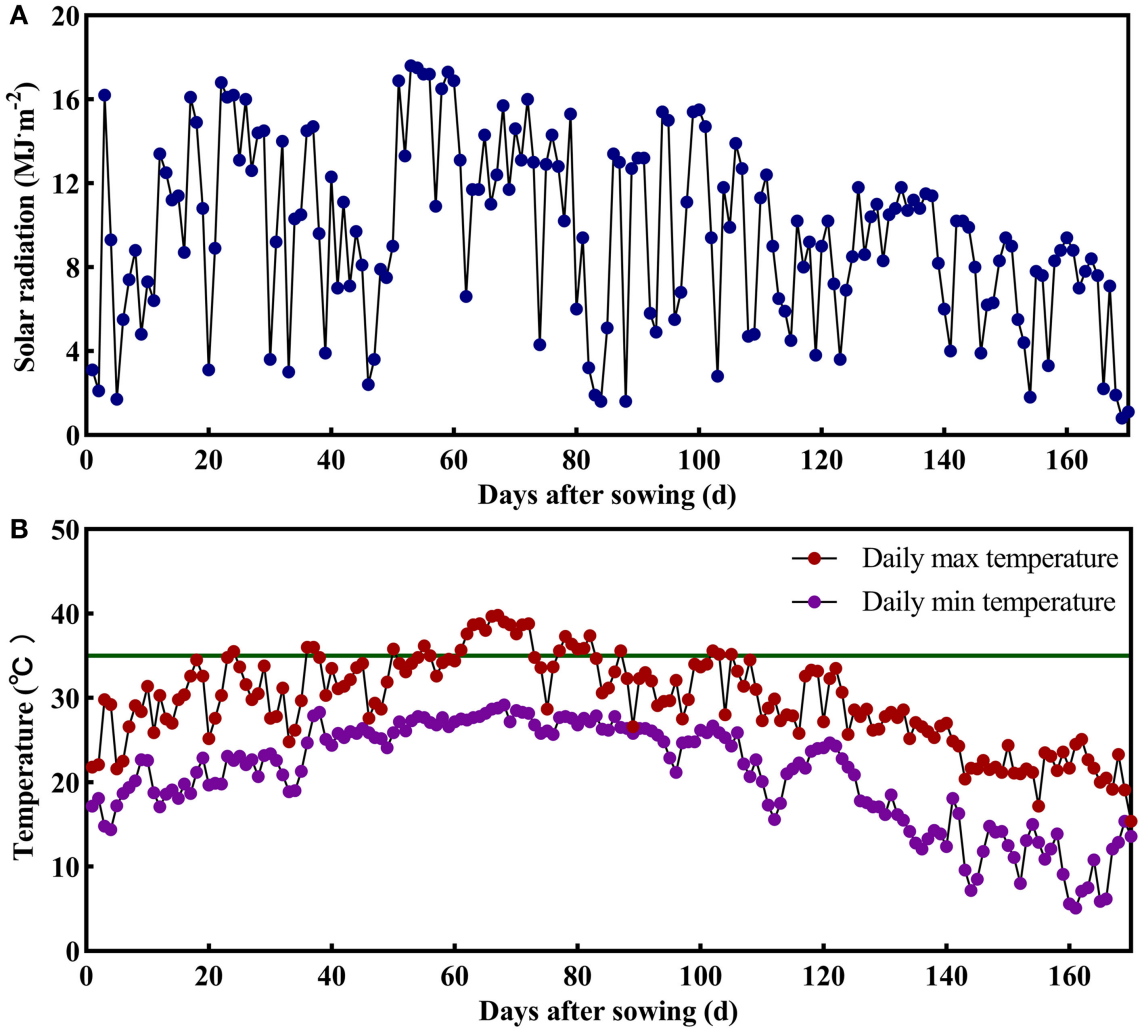
Daily photosynthetically active radiation and daily temperature during the growth period of CJ03 and W1844 at the experiment site of Danyang, Southeast China. The green line indicates a high temperature of 35°C. **(A)**, the daily photosynthetically active radiation. **(B)**, the daily temperature.

The field experiment was conducted in 2018 at Danyang Experimental Base of Nanjing Agricultural University, Jiangsu Province, China (31°54′31″N, 119′28'21″E) during the rice-growing seasons. In order to analyze the poor initiation of IS in large panicle rice, the conducted experiment used two homozygous large panicle japonica rice varieties, namely CJ03 and W1844, from the State Key Laboratory of Rice Genetics and Germplasm Innovation, Nanjing Agricultural University. The agronomic traits are shown in Table 1. Seedlings were field-grown and transplanted 25 days after sowing (May 21, 2018) at a hill spacing of 13.3 × 30 cm with two seedlings per hill. The size of the plot was 7 × 10 m. Each rice variety was grown in three replicate plots in a completely randomized block design. The soil at the experimental site was clay loam. Nitrogen (N) throughout the whole growing season was 280 kg ha^−1^, and the amount of N fertilizer was converted into urea according to the N content. The application ratio of base fertilizer to panicle fertilizer was 5:5. Base fertilizer was applied before transplanting, and the panicle fertilizer was applied when the leaf-age remainder was 3.5. The heading date (50% of plants) for CJ03 and W1844 was from September 1–3 in 2018. Afterward, the plants were harvested from November 5–7 in 2018. The daily photosynthetically active radiation and daily temperature were shown in Figure 1, which was measured during the growth period of CJ03 and W1844 at a weather station close to the experimental site. The cultivation and management measures were applied according to the technical requirements of the local field at the experiment site of Danyang, Southeast China.

**Table 1:** Agronomic traits of CJ03 and W1844.

**Materials**	**Plant height (cm)**	**Panicle length (cm)**	**Grain growth density**	**Grains per panicle**	**1,000-grain weight (g)**	**Seed setting rate(%)**
CJ03	101.12a	21.13a	12.30b	259.42b	20.22b	87.21a
W1844	100.63a	20.67a	13.57a	280.08a	24.53a	85.19b

*Grain growth density = grains per panicle/panicle length. Different letters indicate statistically significant differences at the P = 0.05 level*.

### Experimental Design

A total of 1,600 panicles with similar growth patterns that headed on the same day were chosen and labeled in each replicate. Afterward, the flowering date of each spikelet position of the chosen panicles was observed and identified. On September 2–4, most labeled panicles were withdrawn from the flag leaf sheath completely, the spikelet-thinning treatment was performed according to the protocol shown in Supplementary Figure 1. In total, there were two treatment groups: group one was the control group with no spikelet thinning (labeled as T0); group two had the upper two-thirds of followers removed (labeled as T1) when the IS in the lower part of the panicles is flowering. The primary branches of the whole panicle were divided into three parts according to our previous study (You et al., 2016). These branches were divided accordingly into upper, middle, and lower parts. If the number of primary branches could not be divided equally, a number of spikelets equal to the integer of the average branch number were included in each of the upper and lower parts, and the main parts were included in the middle part. The remaining parts were included in the middle part. The superior spikelets were the grains on the three primary branches of the upper part of the panicle, while the IS were the grains on the three secondary branches in the lower part. The difference in flowering date between SS and IS was almost 4–5 days within a panicle (Supplementary Figure 1).

### Sampling and Measurement

#### Grain Weight and Grain Growth Rate

The experiment involved the sampling of 200 tagged panicles from each replicate plot every 2 days post anthesis (DPA) to 16 DPA (2, 4, 6, 8, 10, 12, and 16 DPA), showing at Supplementary Figure 1. About 3,000 SS and 5,500 IS of tagged panicles in each replicate plot were frozen in liquid N for 1 min before storing at −80°C. These were used for the determination of plant carbohydrate level, starch content, enzyme activities, as well as T6P level and gene expression levels. About 1,000 SS and 1,500 IS of tagged panicles in each replicate plot were deactivated at 105°C for 5 h and dried at 80°C to a constant weight. The grains were then weighed and dehulled to determine the grain dry weight (DW). Grain filling processes were fit to the growth equation as proposed by the study of Richards (Richards, 1959).


(1) $$\begin{array}{r} W=\frac{A}{{\left(1+Be^{-kt}\right)}^{1/N}} \end{array}$$

(2) $$\begin{array}{r} R=\frac{AKBe^{-kt}}{N{\left(1+Be^{-kt}\right)}^{\left(N+1\right)/N}} \end{array}$$

The grain filling rate (R) was calculated as the derivative of Eq. 1, where W is the grain weight (mg), A is the final grain weight (mg), t is the time after anthesis (days), and B, k, and N are coefficients established from the regression of the equation.

#### Endosperm Cells Proliferation Levels

Rice grains of different developmental stages (2, 4, 6, 8, 10, 12, and 16 DPA) were fixed in Kano fixative containing one-fourths of glacial acetic acid, and three-fourths of ethanol at room temperature for 24 h. The method for isolation and counting of endosperm cells was modified by Zhang et al. (1998). The grains were dehulled and passed through 70, 50, and 25% ethanol, then passed into distilled water. The dwell time of each stage ranges from 1 to 12 h. Afterward, the grains were isolated under a dissecting microscope, then they were removed from the embryo with a small insect needle, leaving only the endosperm. Isolated endosperm was stained in Hansen Su Staining Solution (Solarbio, Beijing, China) for more than 24 h and washed several times in distilled water. To completely dissociate the endosperm tissue into a cell suspension, the isolated endosperm was transferred into a 0.1% cellulase solution and bathed in water at a constant temperature of 40°C for more than 4 h. Isolated endosperm cells were diluted to 10 ml by adding 1 ml aliquots to a filter tube containing about 20 ml of distilled water, then passed through a suction filter to sink the cell on the microporous filter. The endosperm cell number in view for each counting chamber was noted using an ordinary microscope.

#### Sucrose, Glucose, Fructose Levels, and Starch Content

The sucrose and starch extraction methods were modified from the method suggested in the study of Yoshida (Yoshida et al., 1976). The grains were firstly frozen in liquid N for 1 min before storing at −80°C and then ground to a fine powder. Approximately 0.1 g of the sample was extracted with 8 ml which has 80% aqueous ethanol at 80°C for 30 min. After cooling, the sample was centrifuged at 5,000 rpm for 15 min and the supernatant was collected in a 50 ml volumetric flask. The extraction process was repeated three times. All the supernatants were combined in the flask with the addition of distilled water to 50 ml. The extract was filtered through a 0.45 μm millipore membrane, and then through the Ultra Performance Liquid Chromatography (UPLC-ELSD) to analyze the sucrose, glucose, and fructose. Conditions of UPLC system (UltiMate™ 3,000, Thermo Scientific™, Germany) were as follows: index detector, ELSD 6,000 (Agilent); column, Shodex sugar column NH2P-504E; column temperature, 30°C; mobile phase, a solvent mixture of acetonitrile and ultra-pure water (75:25 v/v); flow rate, 1 ml/min; and injection volume, 20 μl.

For starch determination, the residue after centrifugation in the tube was oven-dried at 60°C to constant weight, then 2 ml of distilled water was added and put in a boiling water bath for 20 min. Two milliliters of 9.2 mol^**.**^ L^−1^ HClO_4_ was added to the cooled tube and then vortexed for 10 min for complete digestion of starch into glucose. Afterward, the sample was centrifuged at 5,000 rpm for 15 min. The supernatant of the extract was collected in a 50 ml volumetric flask. The extraction process was repeated three times by putting the residue in HClO_4_. Finally, all the supernatants were combined in the flask and distilled water was added up to 50 ml. The starch concentrations were determined with the anthrone method. In a new 15 ml centrifuge tube, 0.1 ml of the extract and 4 ml of 0.2% anthrone were added, then it was placed into an 80°C water bath for 15 min. The colorimetric determination was performed by a chronometer at OD 620 nm.

#### Relative Expression of Genes

Gene transcription levels of the related genes, including *OsSUT1, OsSUT2, OsSUS4, OsAGPL1, OsTPS8*, and *OsSnRK1a*, were analyzed through RNA extraction, cDNA synthesis, and quantitative real-time polymerase chain reaction (qRT-PCR). Tagged grains were sampled every 2 DPA from 2 DPA to 8 DPA, then frozen in liquid N for at least and stored at −80°C for RNA extraction. The RNA-prep pure PLANT Kit (DP432, Tiangen Biotek, Beijing, China) was used to isolate the total RNA from the rice grains, and then the total RNA was reversed-transcribed into the first-strand cDNA with the Prime-Script-TM RT Reagent Kit (RR036, Takara, Kyoto, Japan), oligo-dT. The quantitative real-time polymerase chain reaction was performed using an ABI 7300 sequencer and SYBR Premix Ex Taq-TM (RR420, Takara, Kyoto, Japan) according to the protocol of the manufacturer. All experiments were conducted at least three times, with three samples taken at each time point. The primers used in this research are included in Supplementary Table 1.

#### Determination of Enzymes Activities

The methods testing key enzymes involved in converting sucrose to starch in the grains, the cell wall invertase (CWI), SuSase, and ADP-glucose pyrophosphorylase (AGPase) activities, were measured according to the study of Nakamura (Nakamura et al., 1989). About 120 tagged panicles were sampled from each plot every 2 DPA from 2 to 8 DPA. The samples were frozen in liquid N for 1 min before storing at −80°C. These samples were used to determine the activities of the enzymes. The sampled grains were dehulled and homogenized with a pestle in a precooled mortar containing 5 ml of 50 mM 4-(2-hydroxyethyl)-1-piperazineethanesulfonic acid (HEPES)-NaOH frozen extraction buffer [pH7.5, including 10 mM MgCl_2_, 2 mM ethylenediaminetetraacetic acid (EDTA), 50 mM 2-mercaptoethanol, 12.5% glycerol, and 5% polyvinylpyrrolidone-40 (PVP-40)]. The dehulled and homogenized samples were stored at 0°C. After being filtered through four layers of cheesecloth, the homogenate was centrifuged at 15,000 g for 15 min at 0°C and the supernatant of the crude enzyme extract was used directly for the enzyme assay.

#### Determination of T6P Content

The samples were frozen in liquid nitrogen for 1 min before storing at −80°C, which were used to determine the trehalose-6-phosphate (T6P) level. The T6P levels of the samples were analyzed by plant trehalose-6-phosphate synthase of ELISA Kit (Shanghai Jianglai Biotech, Shanghai, China). Trehalose-6-phosphate synthetase (T6P) level was determined by the double antibody sandwich method. The purified plant trehalose-6-phosphate synthase was used to capture the antibody and coat the microplate to make a solid-phase antibody. Plant T6P was added into the coated microplate in turn, and then combined with Horse Radish Peroxidase (HRP) labeled detection antibody to form antibody-antigen enzyme-labeled antibody complex. After thorough washing, Tetramethylbenzidine (TMB) was added to develop the color. TMB is transformed into blue under the catalysis of the HRP enzyme and yellow under the action of acid. There was a positive correlation between the color and the plant T6P. The absorbance (OD value) was measured at 450 nm by a microplate reader, and the content of T6P was calculated by standard curve.

### Statistical Analyses and Illustration Drawing

The data analyses were employed by using Student's *t*-test. For data presented in bar charts, Duncan's test was conducted to determine differences among the treatments, with statistical significance accepted at *P* < 0.05. Statistical analyses were performed using SPSS Statistics (SPSS Inc, Chicago, IL, USA). Illustrations were drawn in Adobe Photoshop (Adobe, California, USA) and Graph Pad Prism (Graphpad Software Inc., San Diego, USA).

## Results

### Grain Weight and Seed Setting Rate

The two homozygous large panicle japonica rice varieties of CJ03 and W1844 exhibited similar patterns of poor grain filling in IS. The inferior spikelets of the T0 group were poor in gain-filling compared to the IS of the T1 group (Table 2). At maturity, the SS of the T0 group exhibited the highest grain weight and seed setting rate, followed by IS in the T1 group, and IS of the T0 group exhibiting the lowest values (Table 2). In CJ03, the grain weight of IS in the T1 group was lower than SS but significantly higher than the IS of the T0 group; the seed setting rate showed the same pattern. However, the grain weight and seed setting rate on IS of W1844 were higher than the SS of the T0 group after removing spikelets (Table 2), which suggested that the grain growth of IS in W1844 can recover better than CJ03 after removing spikelets.

**Table 2:** Grain weight and seed setting rate of CJ03 and W1844 under different treatments at maturity.

**Materials**	**Treatment**	**Grain weight (mg)**		**Seed setting rate (%)**	
		**Superior**	**Inferior**	**Superior**	**Inferior**
CJ03	T0	23.95a	13.08c	92.07a	84.83c
	T1	–	20.00b	–	87.78b
W1844	T0	27.12a	21.48b	87.96b	84.22c
	T1	–	27.10a	–	93.21a

*T0 represents the control group without any treatment, and T1 represents the top 2/3 of the spikelets that were removed; –,the spikelets that were removed. Different letters indicate statistically significant differences at the P = 0.05 level*.

### Difference in Grain Growth During Early Grain-Filling Stage

**Figure 2:**
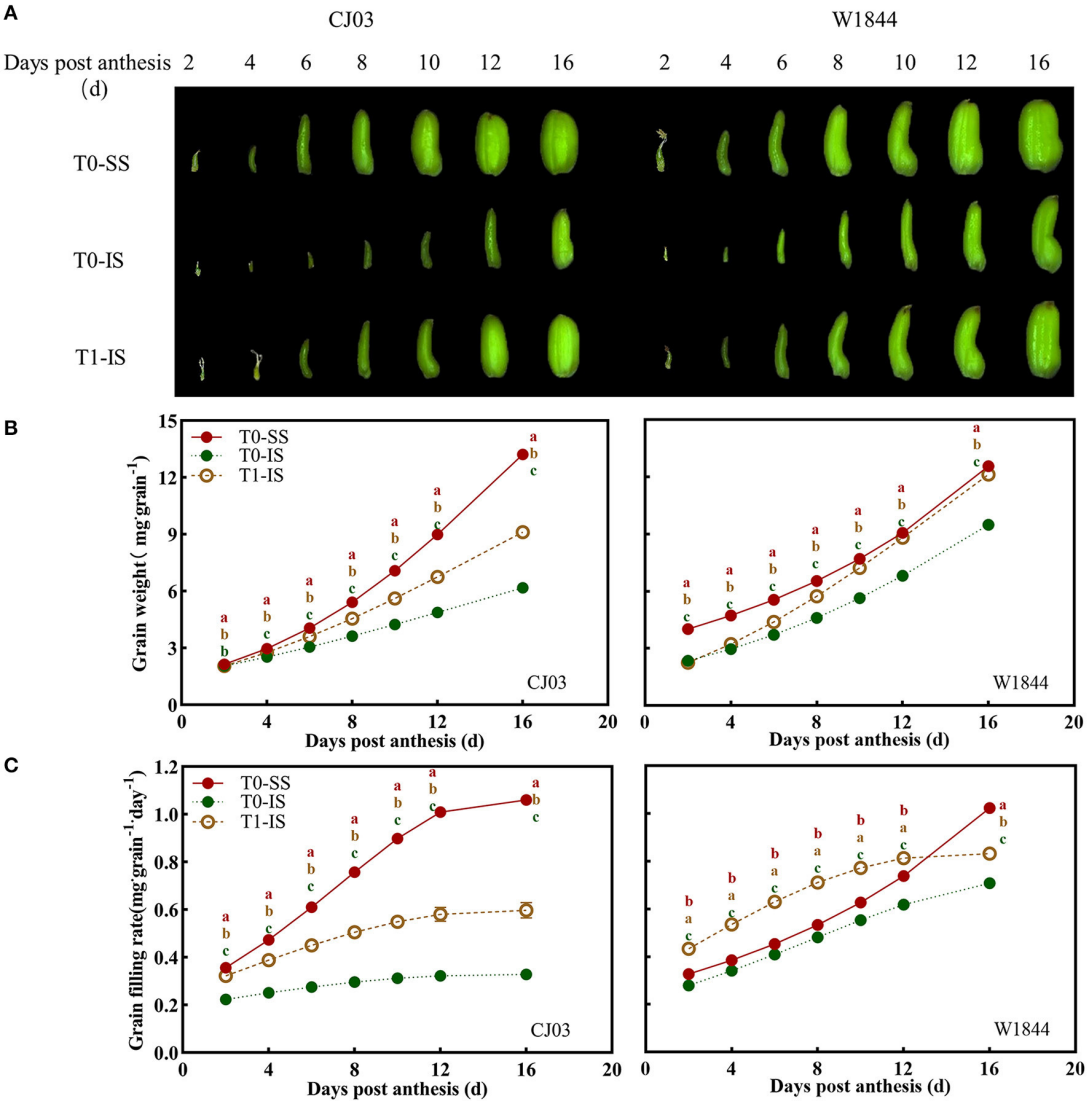
Dynamic of grain growth in CJ03 and W1844 during early grain filling period under spikelet-removing treatments. The T0 and T1 represent the control group without any treatment and the top 2/3 of the spikelets were removed. **(A)**, the morphology of SS and IS in rice during the early grain filling period (observed under stereoscope). **(B)**, the changes of grain weight in the test materials during the early grain filling period under spikelet-removing treatments. **(C)**, changes of grain filling rate in the test materials during early grain filling period under spikelet-removing treatments. Colored letters indicate statistically significant differences between treatments of the same day at the *P* = 0.05 level.

The grain growth in CJ03 and W1844 was assessed during the early grain-filling stage to further confirm the different grain-filling initiation mechanisms between these two varieties (Figure 2). Grains of CJ03 and W1844 grown rapidly in the early grain-filling stage (Figure 2A). The grain-filling initiation of IS in the T0 group was significantly the worst among the IS of the T1 group and SS of the T0 group, while the SS in T0 treatment exhibited the best grain-filling initiation (Figure 2). At 16 DPA, the IS grain weight of the T1 group in W1844 can almost approach the level of SS in the T0 group (Figure 2B). After removing the upper 2/3 spikelets, the grain-filling rate of IS in W1844 was significantly higher than SS from 2 DPA to 12 DPA, whereas the IS of CJ03 was still lower than SS (Figure 2C), indicating that the IS initiation of W1844 can be more effectively improved than that in CJ03.

### Proliferation of Endosperm Cells in Superior and Inferior Spikelets

**Figure 3:**
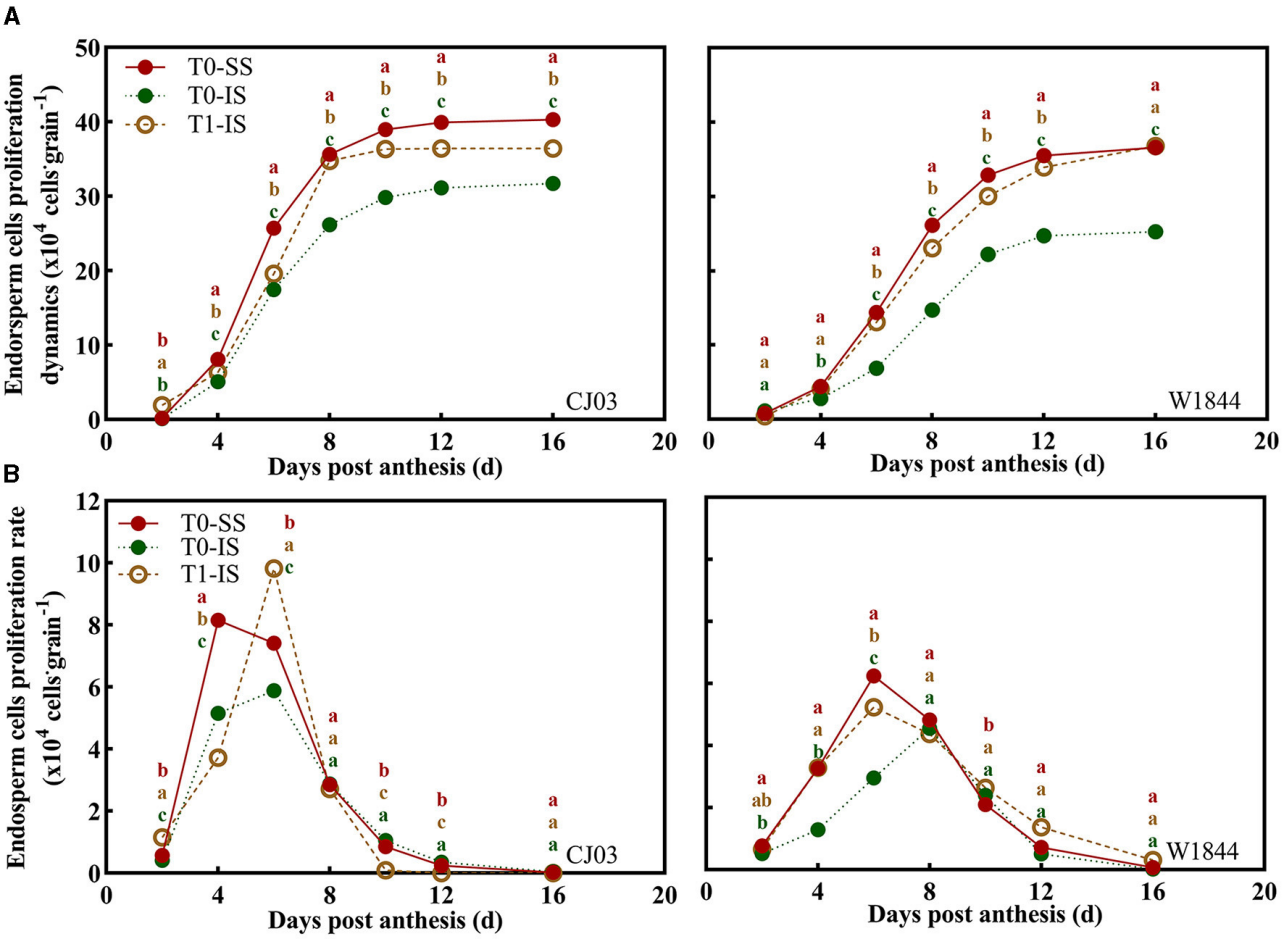
Proliferation changes of endosperm cells in the developing grains of CJ03 and W1844. The T0-control group without any treatment; T1-top 2/3 of the spikelets were removed. **(A)**, the proliferation dynamics of endosperm cells in the test materials during the early grain filling period under spikelet-removing treatments. **(B)**, Proliferation rate changes of endosperm cells in the test materials during early grain filling period under spikelet-removing treatments. Colored letters indicate statistically significant differences between treatments on the same day at the *P* = 0.05 level.

Localization analysis revealed that the proliferation of endosperm cells was altered by different spikelet positions and treatments (Figure 3). The endosperm cells proliferation of SS and IS at 4 DPA began to show a significant difference, which showed the endosperm cell number of SS of the T0 group and IS of the T1 group was significantly more than the IS without removing SS treatment (Figure 3). From 4 to 8 DPA, the endosperm cells of IS divided quickly in CJ03 and W1844, and the maximum proliferation rates of endosperm cells in IS were significantly increased after removing SS in both materials (Figure 3). At 10 to 16 DPA, the IS endosperm cell number in the T1 group was higher than the IS without removing SS, but it was still lower than SS in the T0 group in CJ03 (Figure 3). Nevertheless, the endosperm cell number of IS in the T1 group could reach the level of SS in W1844 (Figure 3), demonstrating that SS removal can be more efficient to improve the endosperm cell proliferation of IS in W1844 than that in CJ03.

### Starch Content and Sucrose-Unloading Ability in Developing Grains

**Figure 4:**
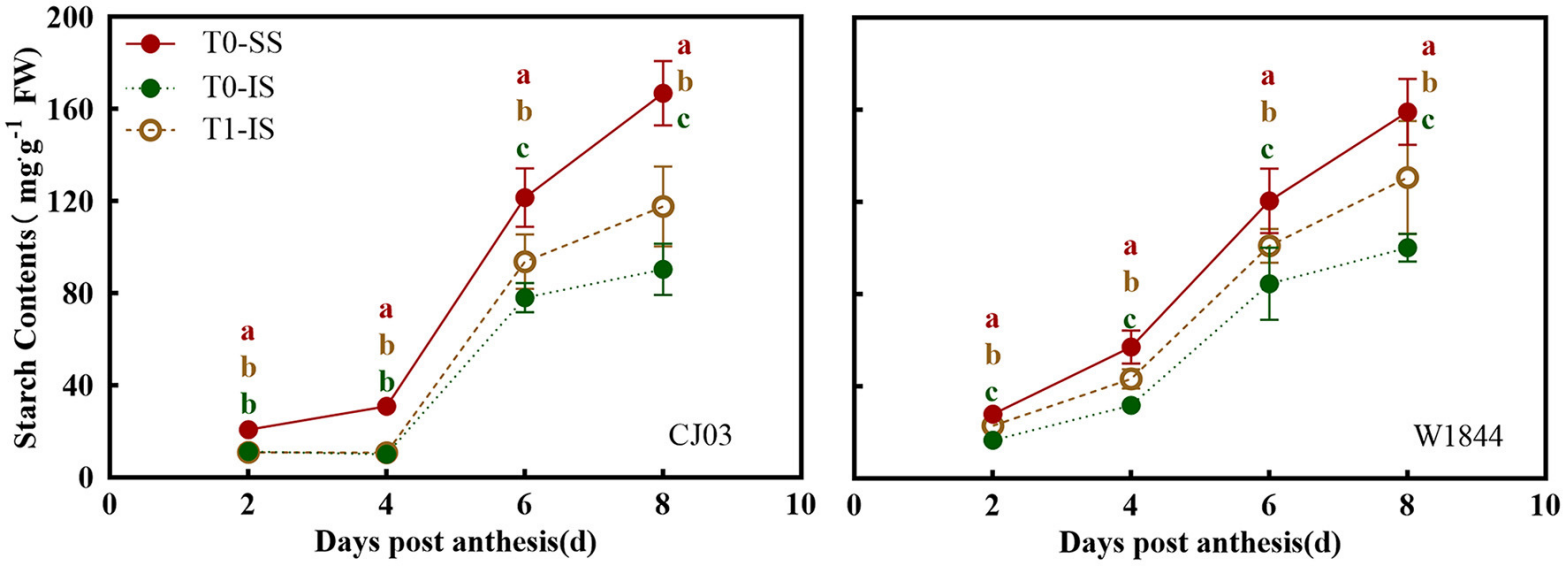
Starch content in developing grains of CJ03 and W1844. The T0-control group without any treatment; T1- top 2/3 of the spikelets were removed. Colored letters indicate statistically significant differences between days of the same treatments at the *P* = 0.05 level.

The starch contents of spikelets in CJ03 and W1844 were examined from 2 to 8 DPA, wherein the removal of the SS of panicles had a positive effect on the starch content of IS (Figure 4). From 6 to 8 DPA, the starch content in IS of both CJ03 and W1844 were significantly increased by spikelets removal. Interestingly, it was shown there was no significant difference in IS of CJ03 between the T0 group and T1 group from 2 to 4 DPA, whereas the IS of W1844 showed a significant difference on that day (Figure 4). This result indicates there are differences existed in starch synthesis ability in CJ03 and W1844.

**Figure 5:**
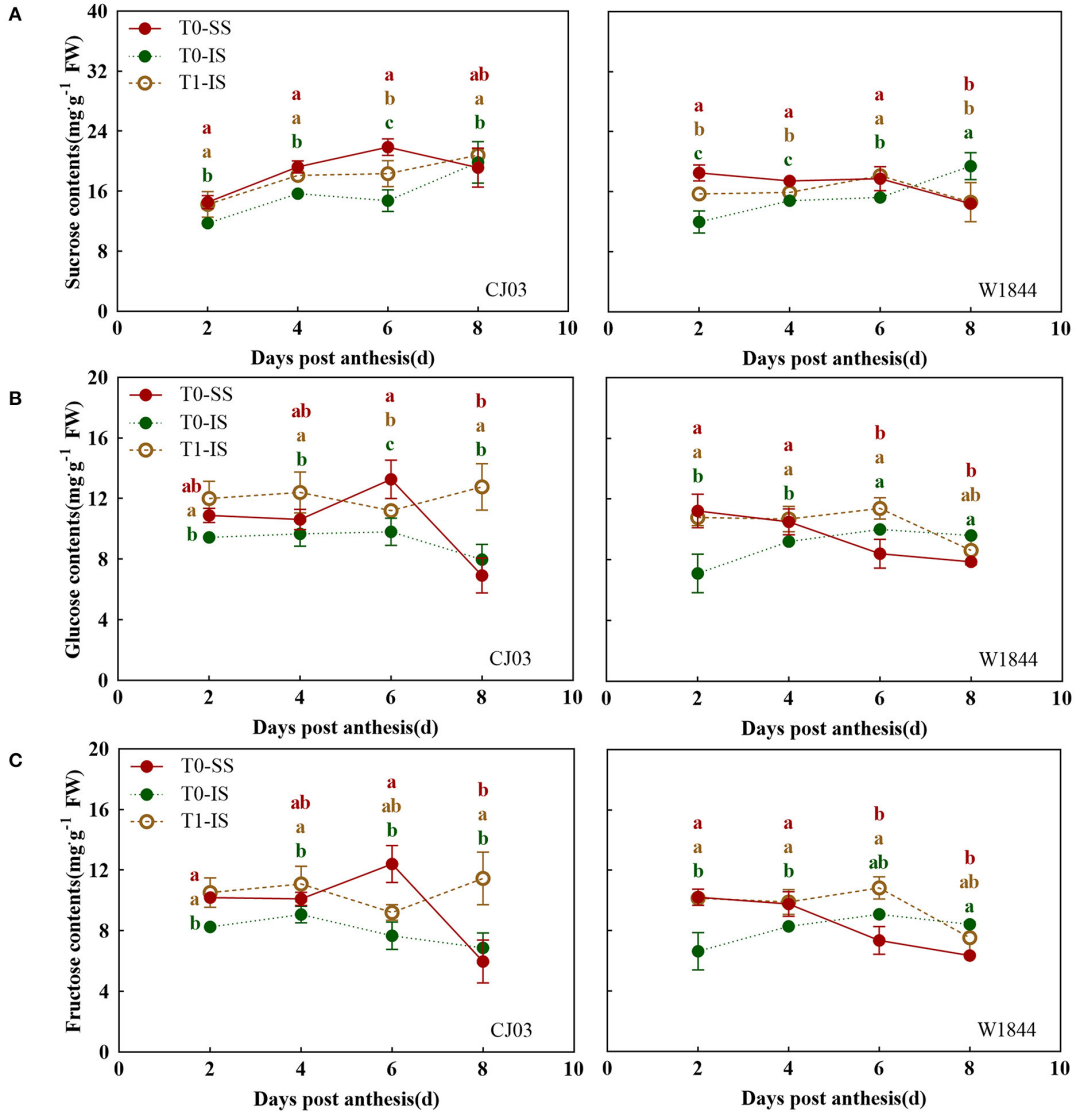
Changes of soluble carbohydrates contents in developing grains of CJ03 and W1844. The T0-control group without any treatment; T1- top 2/3 of the spikelet were removed. Sucrose **(A)**, Glucose **(B)**; and Fructose **(C)** in developing grains of test materials. Colored letters indicate statistically significant differences at the *P* = 0.05 level.

**Figure 6:**
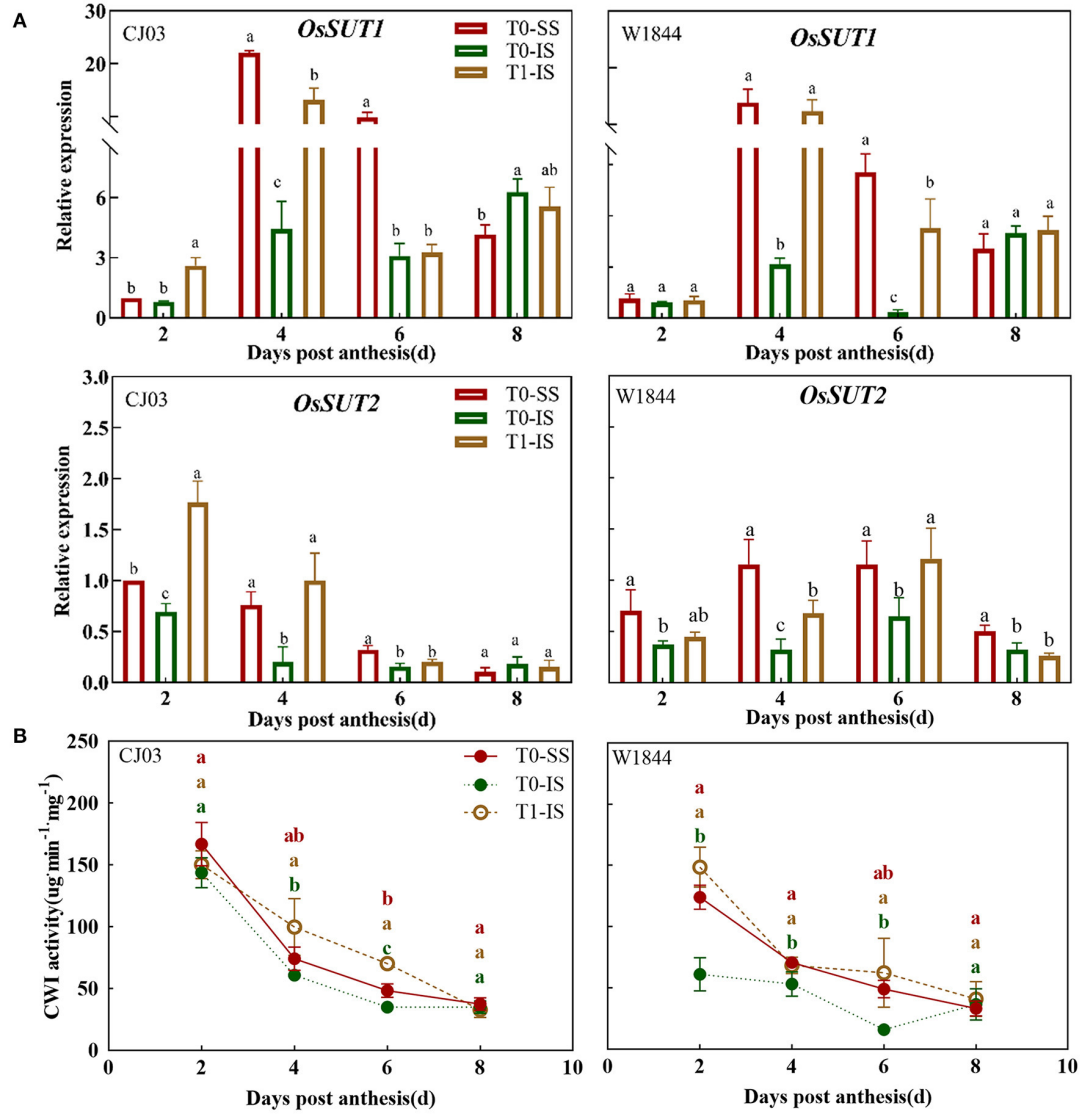
Effects of the conversion of sucrose-unloading in developing grains of CJ03 and W1844 under spikelet-removing treatment. The T0-control group without any treatment; T1- top 2/3 of the spikelets were removed. Red, green, and yellow bars represent the SS of the T0 group, the IS of the T0 group, and the IS of the T1 group. **(A)**, the expression of *OsSUTs* genes was validated by RT-PCR. **(B)**, the CWI activity in SS and IS at the initiation of grain filling period. Colored letters indicate statistically significant differences at the *P* = 0.05 level.

To investigate whether the sucrose-unloading ability is responsible for the difference of starch synthesis, we measured the soluble carbohydrates contents, the expression patterns of *OsSUTs*, and activity of CWI in the grains of different treatments at the early grain-filling stage (0–8 DPA) (Figures 5, 6). The sucrose concentration in IS of the T0 group was nearly the lowest than other treatments from 2 DPA to 6 DPA in both CJ03 and W1844, and the sucrose concentration of IS significantly increased after removing spikelets on that day (Figure 5A). In similar, the hexose concentration in IS of the T1 group was almost higher than the IS of the T0 group from 2 to 6 DPA in both CJ03 and W1844, and even higher than the SS of the T0 group during this period (Figures 5B,C). The expression patterns of *OsSUTs* and activity of CWI in the samples were significantly different between CJ03 and W1844 after spikelets removal (Figure 6). In IS of CJ03, the expression patterns of *OsSUT1* and *OsSUT2* rapidly increased and were even higher than that in the SS by removing spikelets at 2 and 4 DPA (Figure 6A). Similarly, the expression patterns of *OsSUT1* in IS of the W1844 T0 group were significant lowest at 4 DPA and 6 DPA but removing spikelets could significantly increase the expression of *OsSUT1* and *OsSUT2* (Figure 6A). Interestingly, the activity of CWI was obviously increased in IS of CJ03 by removing spikelets from 4to 6DPA, and even higher than SS of the T0 group (Figure 6B). The CWI activity in IS of W1844 significantly increased and was even higher than that in SS by spikelets removal from 2 to 6 DPA (Figure 6B). This result implies that the sucrose unloading ability in IS of the T0 group is low, while the SS removal can obviously improve the sucrose-unloading ability of IS.

### Activities of Key Enzymes and Gene Expression Involved in Metabolism of Sucrose to Starch

**Figure 7:**
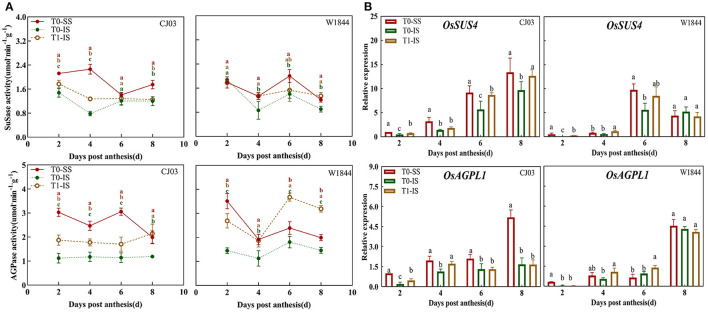
Difference of key enzymes and gene expression involved in of Sucrose to Starch in developing grains of CJ03 and W1844 under spikelet-removing treatments. The T0-control group without any treatment; T1- top 2/3 of the spikelets were removed. Red, green, and yellow bars represent the SS of the T0 group, the IS of the T0 group, and the IS of the T1 group. **(A)**, the activity of SuSase and AGPase in developing grains at the initiation stage of grain-filling. **(B)**, the expression of *OsSUS4* and *OsAGPL1* were validated by RT-PCR. Colored letters indicate statistically significant differences at the *P* = 0.05 level.

A detailed analysis showed there were significant differences in key enzymes and gene expression involving in metabolism and sucrose to starch of different spikelet positions between CJ03 and W1844 (Figure 7). It was shown that the SuSase activity and AGPase activity in IS of W1844 were significantly increased after removing SS, and even higher than that in the SS of the T0 group (Figure 7A). However, those activities of enzymes in IS of the CJ03 T1 group were not significantly higher than the SS of the T0 group in CJ03 (Figure 7A). The expression patterns of *OsSUS4* and *OsAGPL1* in the samples were assessed to further confirm the difference in the metabolism of sucrose to starch (Figure 7B). It was shown that the expression of *OsSUS4* and *OsAGPL1* could be obviously increased in IS after removing spikelets in both two lines (Figure 7B). Interestingly, the expression of *OsSUS4* and *OsAGPL1* were more obviously increased in W1844, which could reach and even higher than the expression of SS from 4 to 8 DPA (Figure 7B). This finding indicates that these changes may cause differential competence of sucrose to starch in IS of the T1 group between CJ03 and W1844.

### Analysis of T6P and SnRK1 Levels

**Figure 8:**
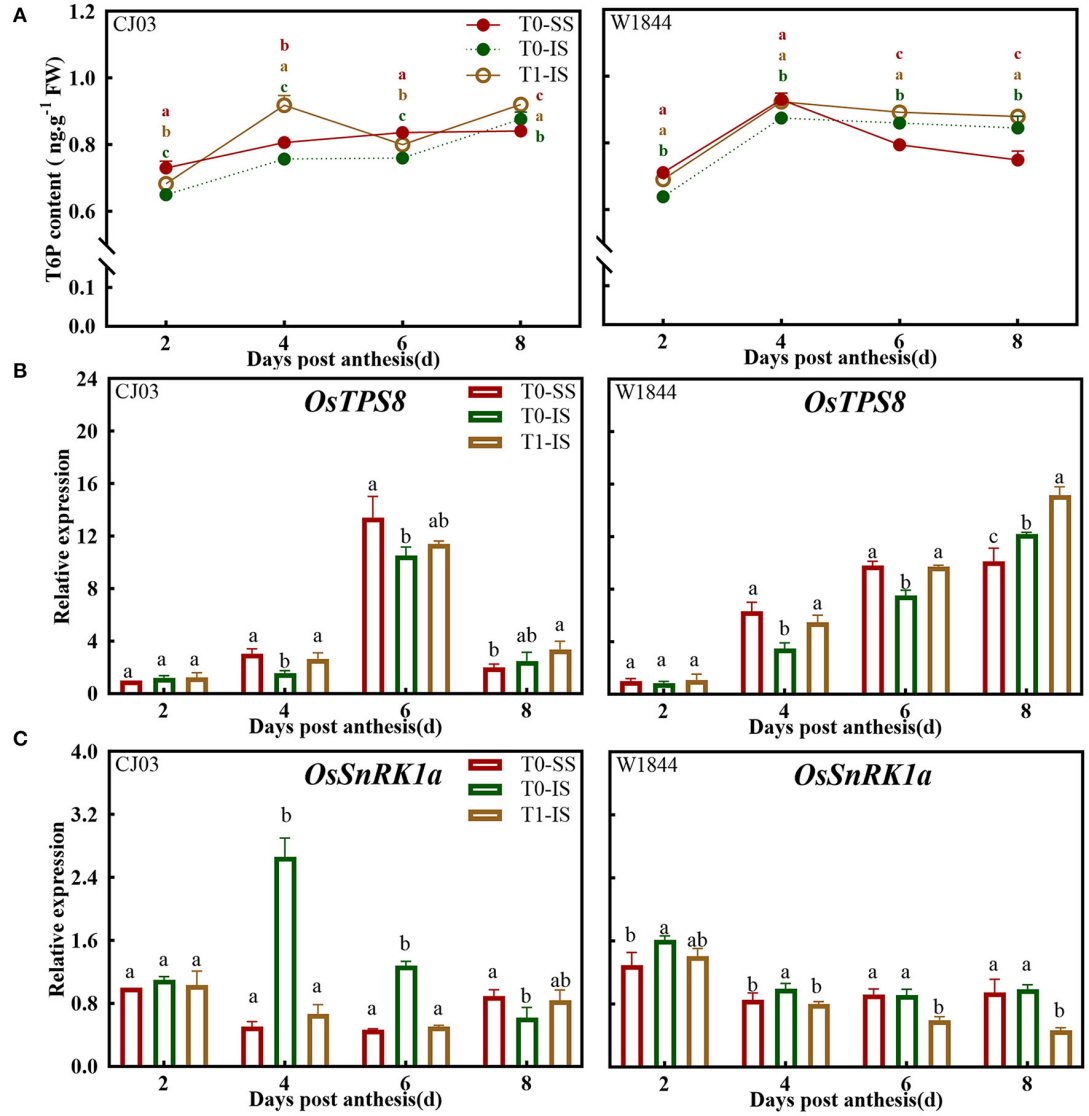
T6P and SnRK1 levels in developing grains of CJ03 and W1844 under spikelet-removing treatment. The T0-control group without any treatment; T1- top 2/3 of the spikelets were removed. Red, green, and yellow bars represent the SS of the T0 group, the IS of the T0 group, and the IS of the T1 group. **(A)**, T6P content in developing grains at the initiation stage of grain-filling. **(B)**, the expression of *OsTPS8* was validated by RT-PCR. **(C)**, the expression of *OsSnRK1a* was validated by RT-PCR. Colored letters indicate statistically significant differences at the *P* = 0.05 level.

Contrasting T6P content and expression pattern of *OsTPS8* and *OsSnRK1a* were obtained over the grain-filling initiation period (Figure 8). The trehalose-6-phosphate concentrations were obviously increased in IS after removing spikelets during the grain-filling initiation period, whereas the only T6P contents in IS of W1844 significantly reached and even higher than the level of SS after removing spikelets (Figure 8A). To increase understanding of the T6P pathway at grain-filling initiation period, the gene expression of *OsTPS8* about T6P synthesis and the gene expression of *OsSnRK1a* about SnRK1 activity were assessed (Figures 8B,C). The expression of *OsTPS8* in IS of the T1 group was significantly increased after removing spikelets from 4 to 8 DPA (Figure 8B). In addition, the expression of *OsSnRK1a* in IS of T1 group was no significantly lower than the SS of the T0 group in CJ03 at grain-filling initiation period, but the expression of *OsSnRK1a* in IS of the T1 group was obviously reach and even lower than the level of SS in W1844 at grain-filling initiation period (Figure 8C). After removing the spikelet, correlations between T6P content and expression of *OsTPS8* and *OsSnRK1a* strongly suggested that the sugar signaling about the T6P-SnRK1 signaling pathway is significantly different in IS of CJ03 and W1844 during the grain-filling initiation period.

## Discussion

### Inferior Grain-Filling Initiation Is Improved Differently by Removing Spikelets in Large Panicle Rice

The phenomena of poor grain-filling and low seed setting rate in IS are common in large panicle rice (Yang, 2010; Fu and Yang, 2012). Commonly, large-panicle rice (*O. sativa* L.) has a long grain-filling stagnation period in IS, which is an important factor restricting grain-filling of IS (Zhang et al., 2015; Chen et al., 2019a). Furthermore, various rice has greater differences in grain-filling of IS after removing spikelets (Wang et al., 2002; You et al., 2016). Interestingly, this study showed a similar phenomenon wherein the grain-filling initiation of IS exhibited distinct differences by removing spikelets in CJ03 and W1844 (Figure 2). The grain-filling initiation of superior spikelets was rapid in both CJ03 and W1844, while the lag phase of grain filling in IS was long in CJ03 and W1844 (Figures 2, 3). As shown previously by researchers (Chen et al., 2019a), the long lag phase in IS of CJ03 and W1844 caused poor grain-filling and low seed setting rate in IS of both two varieties (Table 2). The nutritional hypothesis states that the poor growth of axillary bud is tightly linked with the weak photo-assimilate supply and its metabolic ability (Buskila et al., 2016; You et al., 2016; Wang et al., 2021). By removing the top two-thirds of spikelets of panicles, the grain-filling initiation of IS was significantly improved. However, only the grain-filling initiation of IS in W1844 could recover to the level of SS after removing spikelets, while the IS in CJ03 could not reach the same level (Figures 2, 4). These initial differences led us to hypothesize that eliminating the SS could improve the grain-filling initiation of IS, but there was a different limiting mechanism for grain-filling initiation of IS between CJ03 and W1844.

### Poor Sucrose-Unloading Delays the Grain-Filling Initiation of Inferior Spikelets

After flowering, the spikelets of the panicle may alter its developmental process to rapidly adapt to the growth in rice. Sucrose-unloading, the main modification involved in grain development and grain filling, plays a major role in sucrose supply during the grain-filling period (Eom et al., 2011; Ma et al., 2017). Sucrose deficiency has been proposed to be the major cause of growth restriction for IS in rice, which is a source of carbon skeletons and energy for plant organs useable (Lemoine, 2000; Barbier et al., 2015). The results of this study showed that the grain-filling initiation of SS was quickly in CJ03 and W1844, while the lag time of grain-filling initiation in IS was long in both CJ03 and W1844 (Figure 2). Interestingly, the soluble carbohydrates of IS were low, which was rapidly increased and even higher than the SS by removing spikelets (Figure 5). Additionally, the starch content showed similar changes (Figure 4). Furthermore, the proliferation of endosperm cells and grain-filling initiation in IS of the T1 group significantly got better than the IS of the T0 group (Figures 2, 3). These results could imply that the low supply of soluble carbohydrates was responsible for poor grain-filling initiation in IS, which was associated with the study of Deng (Deng et al., 2021). The grain filling and rice yield were decided on the efficient transport of carbohydrates from the leaves to seeds (sinks) (Chen et al., 2019a), and the *OsSUT1* and *OsSUT2* play an essential role in phloem sucrose-unloading from source to sink tissues in rice (Eom et al., 2011; Ishibashi et al., 2014). Interestingly, the expression of *OsSUTs* and activity of CWI was low in IS of the T0 group, which can reach and even higher than the level of SS after removing spikelets (Figure 6). These findings strongly suggest that the sucrose-unloading is strongly related to the grain-filling initiation of IS in large panicle rice.

### Efficiency of Sucrose to Starch Metabolism Limits the Grain-Filling Initiation of Inferior Spikelets

After the carbohydrates unload to the grains of rice, the sucrose converses to starch through a complex process (Dong and Beckles, 2019). Various type of large-panicle rice frequently fails to exhibit their high yield potential due to poor metabolism of sucrose to starch (Tang et al., 2009; Kato et al., 2015). Some studies have clarified that the metabolism of sucrose to starch was important to the grain-filling of rice (Ishimaru et al., 2003; Zhang et al., 2015). In this study, soluble carbohydrates have been shown to be abundant in the IS by removing spikelets during the grain-filling initiation period (Figure 5), but the starch content of IS was still poor at that period (Figure 4). This phenomenon implied that the metabolic competence of sucrose to starch in IS was poorer than SS. The role of SuSase and AGPase in controlling the metabolism of sucrose to starch are highly related to the grain filling process (Liang et al., 2001). The sucrose synthase is the key enzyme to decompose the sucrose, while the AGPase can regulate the starch synthesis (Jing et al., 2013). The activities and gene expression about these two key enzymes in IS were significantly worse than the SS during the grain-filling initiation period, getting better after spikelet removal (Figure 7). Therefore, the metabolism of sucrose to starch in IS is poorer than SS during the grain-filling initiation period, which can be supported by the observation in the study of You (You et al., 2016). Some researchers have found that soluble carbohydrates can induce the metabolism of sucrose to starch in plants (Lastdrager et al., 2014; MacNeill et al., 2017). Comparative analysis of the key enzymes and gene expression about sucrose to starch metabolism in spikelets of both CJ03 and W1844, revealed that the SuSase (*OsSUS4*) and AGPase (*OsAGPL1*) of IS were significantly up-regulated after removing spikelets (Figure 7). Interestingly, only the IS of W1844 can reach the same and even higher than the level of SS after removing spikelets (Figure 7). Similarly, the grain-filling initiation of IS in the T1 group can recover to the level of SS in W1844, but the initiation of IS still poorer than SS in CJ03 after removing spikelets (Figure 2). These pieces of evidence emphasized that the metabolism of sucrose to starch can be improved by improving the supply of carbohydrates in IS of both CJ03 and W1844, but the efficiency of sucrose to starch metabolism plays a vital part in regulating the grain-filling initiation of IS.

### Sugar Signaling May Be Responsible for Sucrose to Starch Metabolism of Inferior Grain-Filling Initiation

The carbohydrates can act as sugar signaling to control the process like carbohydrate metabolism, sucrose transport, among others (Jy et al., 2020; Liao et al., 2020). As one of the key sugar-signaling molecules, T6P is central for efficient sucrose utilization (Griffiths et al., 2016; Zl et al., 2021). The T6P pathway is positively related to the supply of soluble carbohydrates in plants, which is strongly connected with the ability of sucrose to starch metabolism (Kolbe et al., 2005; Griffiths et al., 2016). Furthermore, the T6P level can be regulated by trehalose-6-phosphate synthase (TPS), and *OsTPS8* takes part in encoding the TPS enzyme (Zang et al., 2011; Fichtner and Lunn, 2021). Interestingly, T6P regulates the carbohydrate metabolism *via* the SnRK1 pathway in inhibiting the activity of SnRK1, while the SnRK1 protein kinase negatively modulate the growth of plants (Delatte et al., 2011; Lin et al., 2014). The *OsSnRK1a* is one of the SnRK1 α-subunit genes, functioning in the sugar signaling cascade (Lu et al., 2007; Filipe et al., 2018). Consistent with this proposal, the sucrose-unloading ability obviously increased and even can reach the level of SS after removing spikelets (Figure 6). Furthermore, the supply of soluble carbohydrate in IS of the T1 group was obviously increased during the grain-filling initiation period (Figure 5). Similarly, the T6P content and the expression of *OsTPS8* were obviously increased in IS of the T1 group, and the expression of *OsSnRK1a* was decreased to a certain degree (Figure 8). These data suggest that the sugar signaling is strongly connected with the supply of soluble carbohydrates during the grain-filling initiation period of IS in rice. As discussed in the previous sections, the metabolism competence of sucrose to starch in IS of W1844 was higher than CJ03, which was significantly different in the activity of key enzymes and gene expression about the metabolism of sucrose to starch (Figure 7). The key enzymes are related to the metabolism of sucrose to starch which is regulated by the T6P and/or SnRK1 signaling pathway (Kolbe et al., 2005; Chen et al., 2019c). Interestingly, the sugar signaling about the T6P-SnRK1 signaling pathway is significantly different in IS of CJ03 and W1844 during the grain-filling initiation period (Figure 8). As shown by previous researchers (Zhang et al., 2015; Tao et al., 2021), these data identified a novel role that the regulation of sugar signaling may be responsible for the metabolism efficiency of sucrose to starch in the grain-filling initiation of IS in large panicle rice.

## Conclusions

**Figure 9:**
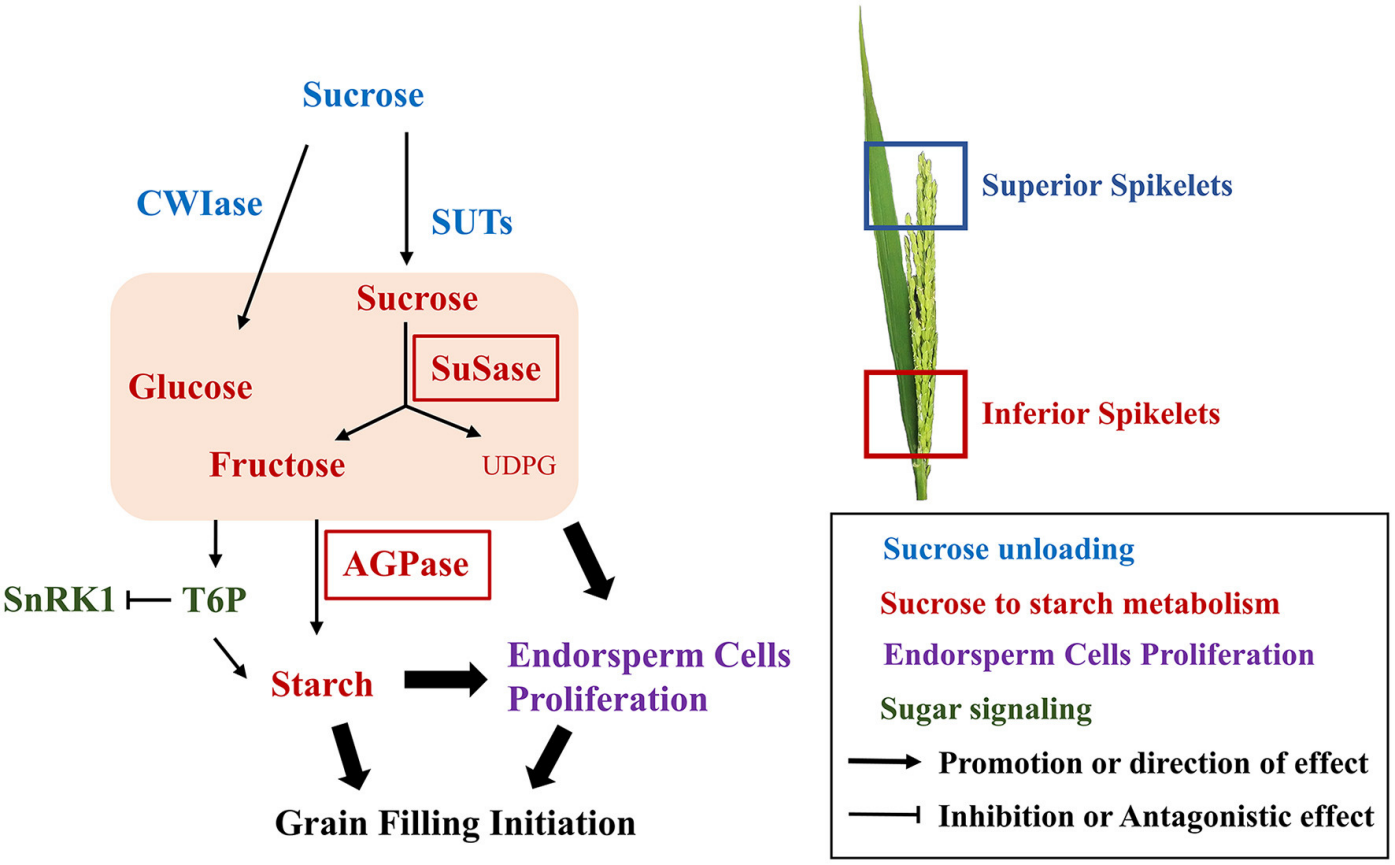
Biochemical pathway to the grain-filling initiation. Solid arrows direction effects; broken line indicates the indirection of effect; different colors indicate various metabolism.

In this study, we compared the grain-filling initiation in CJ03, which has high sink capacity but poor initiation of inferior grain filling, with W1844. Examination of the initiation process of grain filling revealed the poor sucrose-unloading ability was obviously improved in both CJ03 and W1844 after removing spikelets. However, there was a significant difference in the efficiency of sucrose to starch metabolism and led to the poor grain-filling initiation in IS of CJ03. Further studies in sugar signaling of T6P and SnRK1 levels were conducted to prove this hypothesis (Figure 9). An improved understanding of the physiology and biochemistry responses to initiation of grain-filling will help to increase rice productivity and quality.

## Data Availability Statement

The raw data supporting the conclusions of this article will be made available by the authors, without undue reservation.

## Author Contributions

ZJ and GL designed the experiments. ZJ, LC, QC, HY, and MZ conducted the experiment. ZJ and GL analyzed the data and wrote the manuscript. YD, WL, ZL, YJ, and GL revised the manuscript. All authors read and approved the final manuscript.

## Funding

This work was supported by the National Key Research and Development Program of China (2018YFD0300803 and 2017YFD0301204), the National Natural Science Foundation of China (31871573 and 31901454), the Natural Science Foundation of Jiangsu Province for Excellent Young Schloars (BK20200079), and the Jiangsu Agriculture Science and Technology Innovation Fund [CX(18)1002].

## Conflict of Interest

The authors declare that the research was conducted in the absence of any commercial or financial relationships that could be construed as a potential conflict of interest.

## Publisher's Note

All claims expressed in this article are solely those of the authors and do not necessarily represent those of their affiliated organizations, or those of the publisher, the editors and the reviewers. Any product that may be evaluated in this article, or claim that may be made by its manufacturer, is not guaranteed or endorsed by the publisher.
