# Supplementary material for: Efficiency of Sucrose to Starch Metabolism Is Related to the Initiation of Inferior Grain Filling in Large Panicle Rice
Source: Front Plant Sci. 2021 Sep 13;12:732867. doi: 10.3389/fpls.2021.732867 (PMC8473919; doi:10.3389/fpls.2021.732867)
Supplement: Supplementary file 1 [file Data_Sheet_1.DOCX]

Supplementary Material

# Supplementary Figures and Tables

## Supplementary Table

**Supplemental Table S1. Sequences of primers for Actin and sucrose transporter genes for qRT-PCR.**

| Gene | Forward primer 5′ → 3’ | Reverse primer 5′ → 3’ |
| --- | --- | --- |
| *Actin* | CAATCGTGAGAAGATGACCC | GTCCATCAGGAAGCTCGTAGC |
| *OsSUT1* | GCTTTCAACCAGGGTGTCAG | ACTTTCCGGCACATTGGTTC |
| *OsSUT2* | TCTTTTATCGGTGGGCTGGT | TTGCAAAGAATGGCCGACAA |
| *OsSUS4* | TCCGTGAACTGGCGAAGACT | CCCAAGTTCGTCACTTGCTG |
| *OsAGPL1* | GGAAAGGTTCCTATTGGAATCG | GGAGGGCTTTATTCCACCTCAG |
| *OsTPS8* | TTAATCCTCAGGGTGTGGGC | CAGGCGAAGGGCTGATCATA |
| *OsSnRK1a* | AACCAGAGGTAACAGGCAGG | CATCTGTCAAGGAATGCAGG |

##
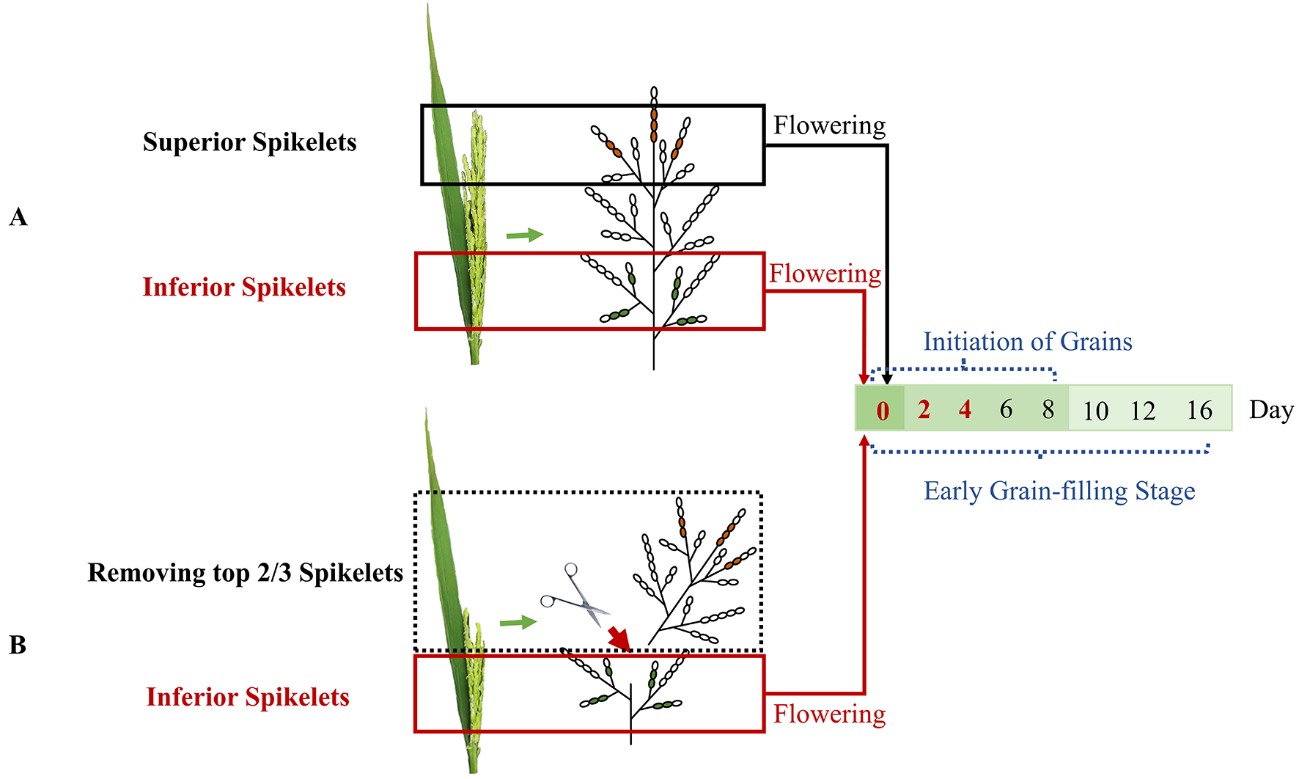
Supplementary Figure

**Supplementary Figure S1. Schematic representation of rice CJ03 and W1844 applied to study the effects of flowering time gaps and competition between rice panicles on different positions.** (A), the control group with no spikelet thinning (labeled as T0), the Black arrow shows the flowering date in superior spikelets (SS) of rice on T0 treatment, and the red arrow shows the flowering date in Inferior spikelets (IS) which was almost 4-5 days after the flowering date of SS of rice on T0 treatment; the number on green frame indicate the dates of sampling for rice in SS and IS. (B), the upper 2/3 followers removed (labeled as T1) when the IS in the lower part of the panicles is flowering, and the red arrow shows the flowering date in IS of rice on T1 treatment, the number on the green frame indicate the dates of sampling for rice in IS.
